# Factors influencing return to work after hip and knee arthroplasty

**DOI:** 10.1186/s10195-018-0515-x

**Published:** 2019-01-14

**Authors:** Lorcan McGonagle, Lila Convery-Chan, Penelope DeCruz, Samantha Haebich, Daniel P. Fick, Riaz J. K. Khan

**Affiliations:** 1 Geraldton Regional Hospital, 51-85 Shenton Street, Geraldton, WA 6530 Australia; 2 University of Western Australia, 35 Stirling Hwy, Crawley, WA 6009 Australia; 3 The Joint Studio, Hollywood Medical Centre, Suite 1, 85 Monash Avenue, Nedlands, WA 6009 Australia; 4 Department of Medicine, Notre Dame University, 32 Mouat St, Fremantle, WA 6160 Australia

**Keywords:** Return to work, After, Hip, Knee, Arthroplasty

## Abstract

**Background:**

A substantial proportion of patients undergoing lower limb arthroplasty are of working age. This study aims to identify when patients return to work (RTW) and if they return to normal hours and duties, and to identify which factors influence postoperative RTW. The hypothesis is that there is no difference in time of RTW between the different types of surgery, and no difference in time of RTW based on the physical demands of the job.

**Materials and methods:**

Consecutive patients aged < 65 years who had undergone unilateral primary total hip arthroplasty (THA), total knee arthroplasty (TKA), or medial unicompartmental knee arthroplasty (UKA) from 2015 to 2017 were sent a questionnaire. Quantitative questions explored timing and nature of RTW, and qualitative questions explored factors influencing timing of RTW.

**Results:**

There were 116 patients (64 male, 52 female) with an average age of 56 years. Thirty-one patients were self-employed and 85 were employees. Of these patients, 58 had undergone THA, 31 had undergone TKA, and 27 had undergone UKA. One hundred and six (91%) patients returned to work. Patients returned to work after (mean) 6.4 weeks (THA), 7.7 weeks (TKA), and 5.9 weeks (UKA). Time of RTW was not significantly influenced by type of surgery (*p* = 0.18) (ns). There was a non-significant correlation between physical demands of the work versus time of RTW (*p* = 0.28) (ns). There was a significantly earlier time of RTW if flexible working conditions were resumed (*p* = 0.003). Active recovery, motivation, necessity and job flexibility enabled RTW. The physical effects of surgery, medical restrictions and work factors impeded RTW.

**Conclusion:**

The time of RTW was not significantly influenced by the type of operation or by the physical demands of the job. Patients returned to work 5.9–7.7 weeks after hip/knee arthroplasty. Rehabilitation, desire, and necessity promoted RTW. Pain, fatigue and medical restrictions impeded RTW.

**Level of evidence:**

3.

## Introduction

A substantial proportion of patients undergoing lower limb arthroplasty surgery are of working age. The United Kingdom National Joint Registry showed that patients aged < 65 years at the time of surgery account for 40% of all total hip arthroplasty (THA) procedures and 32% of all knee arthroplasty procedures [1]. The overall number of lower limb arthroplasty operations is increasing. For example, in 2016, hip arthroplasties increased by 3.7%, and knee arthroplasties by 3.5% according to the Australian Orthopaedic Association National Joint Replacement Registry [2].

Most patients successfully resume employment within a year of their joint arthroplasty with rates as high as 87% and 85% for THA and total knee arthroplasty (TKA), respectively [3]. The time of return to work (RTW) has been shown to vary from 1 to 14 weeks after hip arthroplasty and 8 to 12 weeks after knee arthroplasty [4]. Patients and surgeons are unsure about the optimal time to RTW after arthroplasty. However, there is limited research investigating factors that influence RTW after arthroplasty.

The primary aims of this study were to identify when patients return to work and if they return to normal working hours and duties after lower limb arthroplasty.

The secondary aim was to identify which factors influence postoperative RTW.

The hypothesis is that there is no difference in time of RTW between the different types of surgery, and no difference in time of RTW based on the physical demands of the job.

## Materials and methods

A mixed methodology was used in this study to explore factors influencing RTW for patients after lower limb arthroplasty. A quantitative approach sought to determine timing of RTW, and nature of RTW with regard to hours/duties. The qualitative approach sought a deeper understanding of patient perspectives and experiences. Ethical approval was received from the institutional review board. All operations were conducted in a private metropolitan hospital in Western Australia, and performed by one of the two senior authors.

Consecutive patients undergoing unilateral primary THA, TKA, or medial unicompartmental knee arthroplasty (UKA) from 2015 to 2017 were identified from the research database of the senior authors. Patients meeting the criteria below were invited to complete the questionnaire.

Inclusion criteria were aged ≤ 65 years at the time of surgery (at 65 years one is eligible for an age-related pension in Australia) [5]; surgery conducted within the past 6–12 months; primary THA, TKA or UKA; and engaged in paid work in the 3 months prior to surgery. Exclusion criteria were revision hip/knee arthroplasty and bilateral concurrent hip/knee arthroplasty.

A 13-item questionnaire was developed (Appendix 1). A combination of categorical level questions and free text response questions were included. The questionnaire collected data on patient demographics, employment status, expected and actual number of weeks when work resumed after surgery, operation type, duties and hours initially resumed relative to the usual ones, and perception on appropriateness of the time taken to RTW. Free text responses required patients to describe what helped them RTW after surgery and what limited or prevented them from returning sooner.

RTW was defined as the resumption of paid employment at any capacity. Time was measured in weeks. Patients responded as to how frequently they adopted various postures, and moved loads during a typical workday. The level of physical demand of the individual’s job was then classified by a researcher as per a modified metric version of the Dictionary of Occupational Titles classification, as shown in Table 1 [6].

**Table 1:** Descriptions of work classification [6]

Sedentary work	Exerting up to 10 lb of force occasionally (occasionally: activity or condition exists up to 1/3 of the time) and/or a negligible amount of force frequently (frequently: activity or condition exists from 1/3 to 2/3 of the time) to lift, carry, push, pull, or otherwise move objects, including the human body. Sedentary work involves sitting most of the time, but may involve walking or standing for brief periods of time. Jobs are sedentary if walking and standing are required only occasionally and all other sedentary criteria are met
Light work	Exerting up to 20 lb of force occasionally, and/or up to 10 lb of force frequently, and/or a negligible amount of force constantly (constantly: activity or condition exists 2/3 or more of the time) to move objects. Physical demand requirements are in excess of those for sedentary work. Even though the weight lifted may be only a negligible amount, a job should be rated light work: (1) when it requires walking or standing to a significant degree; or (2) when it requires sitting most of the time but entails pushing and/or pulling of arm or leg controls; and/or (3) when the job requires working at a production rate pace entailing the constant pushing and/or pulling of materials even though the weight of those materials is negligible. The constant stress and strain of maintaining a production rate pace, especially in an industrial setting, can be and is physically demanding of a worker even though the amount of force exerted is negligible
Medium work	Exerting 20 to 50 lb of force occasionally, and/or 10 to 25 lb of force frequently, and/or greater than negligible up to 10 lb of force constantly to move objects. Physical Demand requirements are in excess of those for light work
Heavy work	Exerting 50 to 100 lb of force occasionally, and/or 25 to 50 lb of force frequently, and/or 10 to 20 lb of force constantly to move objects. Physical demand requirements are in excess of those for medium work
Very heavy work	Exerting in excess of 100 lb of force occasionally, and/or in excess of 50 lb of force frequently, and/or in excess of 20 lb of force constantly to move objects. Physical demand requirements are in excess of those for heavy work

The questionnaire was sent to patients aged <65 years who had undergone THA, TKA, or UKA from 2015 to 2017; it was sent electronically to those with a valid email address or by post to those without. The questionnaire was sent to 250 patients and 160 (64%) patients responded. Of these respondents, 41 (25.6%) had not been employed in the 3 months prior to surgery and were therefore excluded from the analysis. Three surveys were incomplete and deemed invalid. The remaining 116 (72.5%) respondents met the eligibility criteria.

Descriptive and categorical data were collated to a MS Microsoft excel spreadsheet and descriptive statistics applied to derive counts and percentages. The qualitative content analysis was conducted through systematic appraisal of the free text responses to identify common themes. A statistician analysed the quantitative data for statistical significance. Multiple regression analysis was performed to assess time of RTW versus operation and flexible working conditions, respectively. Job categories were combined when assessing flexible working conditions due to low numbers in the heavy and very heavy categories. Spearman’s rank order correlation was performed to assess time of RTW versus physical demands of the job.

Two researchers, an orthopaedic surgeon and a qualitative researcher, performed two independent analyses of the data. There was good agreement between the two independent analyses of the data in identifying the main factors relevant to RTW after hip and knee arthroplasty.

## Results

There were 116 patients (64 male, 52 female) with an average age of 56 years (± 7.3SD). Thirty-one patients (26.7%) were self-employed and 85 (73.3%) were employees. Preoperatively, patients worked an average of 41 h per week. Table 2 outlines the physical demands of the jobs and the number of patients. Of the 116 patients, 58 had undergone THA, 31 had undergone TKA, and 27 had undergone UKA.

**Table 2:** Physical demands of job

Physical demands of job	No. of patients	Percentage of patients
Sedentary	36	31
Light	45	38.7
Medium	18	15.5
Heavy	12	10.3
Very heavy	5	4.3

### Time of RTW

One hundred and six patients (91.4%) returned to work, and 10 (8.6%) patients did not. Five (4.3%) cited retirement as the reason, 3 (2.6%) reported physical health reasons, and 2 (1.7%) had been made redundant.

The average time of RTW was 6.4 ± 3.8 weeks for THA, 7.7 ± 3.9 weeks for TKA, and 5.9 ± 3.2 weeks for UKA. Table 3 outlines the physical demands of the jobs versus operation versus time of RTW.

**Table 3:** Operation versus work load versus return to work time

Operation	Work type	Return to work time (weeks ± standard deviation)
THR	Sedentary	5.5 ± 3.8
	Light	7 ± 3.1
	Medium	7.6 ± 3.1
	Heavy	5.8 ± 3.8
	Very heavy	6.3 ± 4
TKR	Sedentary	6.3 ± 2.4
	Light	7.6 ± 4
	Medium	7.3 ± 3.4
	Heavy	9.7 ± 1.5
	Very heavy	12 ± N/A
UKR	Sedentary	6 ± 2.4
	Light	8.8 ± 5
	Medium	3.2 ± 2.1
	Heavy	4.5 ± 1.5
	Very heavy	12 ± N/A

A multiple regression analysis was performed and the time of RTW was not significantly influenced by operation type (*p* = 0.18) (ns). A Spearman’s rank-order correlation found a non-significant correlation between physical demands of the work versus time of RTW (*p* = 0.28) (ns).

Multiple regression analysis also identified a significantly earlier time of RTW if flexible work conditions were resumed (*p* = 0.003). Furthermore, those in less physically demanding jobs (sedentary, light, medium) were more likely to RTW with unchanged conditions of employment compared to those with more physically demanding jobs (heavy, very heavy) (49.5% vs 11.8%). Those with more physically demanding jobs were more likely to RTW with both reduced hours and reduced level of duties (52.9% vs 16.2%).

Thirty-five (30.2%) patients resumed ‘lighter’ duties, whilst 71 (61.2%) patients returned to usual duties. Forty-five (38.8%) patients resumed fewer hours per week, whilst 61 (52.6%) patients resumed their usual hours of work.

Two (1.7%) statistical outliers (time of RTW more than three standard deviations above the mean) were removed from the analysis. One respondent (THA) took long service leave and returned at 26 weeks, and the other (UKA) took 20 weeks to find a new job.

Sixty-eight (58.6%) patients cited the doctor as the source of advice on returning to work. Other sources included their physiotherapist, other patients, and the internet. Eighty-six (74.1%) patients felt the amount of time of RTW was about right, whilst 12 (10.3%) patients reported returning too early and 8 (6.9%) patients believed they could have been back at work earlier.

### Factors enabling RTW

The responses for THA, TKA and UKA are grouped together. A summary of the responses is presented in Table 4).

**Table 4:** Categorisation of theme responses

	Total number	% of total	Operation			Job status		Duties resumed		Hours resumed		Reflection on time taken to RTW		
			THR	TKR	UKR	Self employed	Employee	Lighter	Usual	Fewer	Usual	Too early	About right	Too late
All respondents	116	100	58	31	27	31	85	35	71	45	61	12	86	8
Theme														
Motivations														
Active recovery	71	61	36	17	18	14	57	22	49	27	44	6	61	4
Psychological factors	24	21	14	5	5	6	18	7	17	8	16	4	16	4
Necessity	12	10	8	4	0	5	7	4	8	5	7	3	9	0
Job flexibility	12	10	5	3	4	6	6	4	8	7	5	2	10	1
Barriers														
Physical effects of surgery	44	38	17	16	11	15	29	14	27	22	19	5	33	3
Employment factors	26	22	17	3	6	6	20	5	14	5	14	4	14	1
Medical restrictions	20	17	12	3	5	5	15	8	12	9	11	2	16	2

*Active recovery*: walking, cycling, hydrotherapy, formal physiotherapy sessions

*Psychological factors*: personal attributes, desire to work, escape boredom, discipline, motivation, determination, desire, willingness, return to normal routine

*Necessity*: financial need, self employed, starting new job, meeting deadlines, no leave left

*Job flexibility*: work remotely, reduced hours, self employed

*Physical effects of surgery*: pain, fatigue, weakness, joint swelling, mobility problems

*Employment factors*: inability to physically perform job, no light duties, workplace holiday closures, stairs but no elevators, no place to elevate operated limb

*Medical advice*: medical advice regarding returning to work/driving, inability to drive

Active recovery (e.g., walking/cycling) and formal physiotherapy sessions were key enablers for 71 (61.2%) patients. Psychological factors such as a desire to RTW and escape boredom enabled 24 (20.7%) patients to RTW. The necessity to RTW for pragmatic reasons, e.g., financial need was reported by 12 (10.3%) patients. Job flexibility with work arrangements and hours was a positive influence on the ability of 12 (10.3%) patients to RTW.

### Factors impeding RTW

Sixteen (13.8%) patients reported having had no barriers or limitations on their ability to RTW.

The physical effects of surgery, e.g., pain for 33 (28.4%) patients and fatigue for 10 (8.6%) patients, were the main barriers to RTW.

Work-related factors impeded RTW in 26 (22.4%) patients. Impaired ability to perform work duties was reported by 14 (12.1%) patients, with physically demanding duties being cited as the most common reason.

Twenty (17.2%) patients were limited by medical advice or clearance dictating when they could RTW and drive.

## Discussion

In our study, 91.4% of patients returned to work after arthroplasty. Time of RTW was 5.9 weeks for UKA, 6.4 weeks for THA, and 7.7 weeks for TKA. Rehabilitation, desire, and necessity enabled RTW. Pain, fatigue and medical restrictions impeded RTW.

Foote et al. [7] reported a high number of patients (82%) returning to work after UKA/TKA. There was no significant difference in the ‘physical intensity’ of preoperative occupation versus postoperative occupation. However their cohort did have a longer time of RTW—11 weeks for UKA and 12 weeks for TKA.

In a study by Lyall et al. [8], 40/41 (97.6%) patients aged < 60 years who were working preoperatively returned to work after TKA. They returned to the same work, with no reduction in work intensity. The average time of RTW was 10 weeks. Of their patients, 30/41 (73.2%) were in ‘non manual’ jobs, which is comparable to the 69.8% of patients in our study in sedentary/light jobs.

Jorn et al. [9] combined data on patients aged < 60 years undergoing UKA/TKA; 52/88 (59.1%) of their patients returned to work postoperatively. In contrast to our study, they found that a preoperative light workload was associated with a shorter postoperative sick leave than a medium or heavy workload.

Our study showed that 45/116 (38.8%) patients worked fewer hours when returning to work. Tilbury et al. [10] noted a significant decrease in working hours at 1 year postoperatively in THA and TKA in patients aged < 65 years.

Our study had quicker times of RTW than most other studies. This is likely due in part to 30.2% of patients taking on lighter duties and 38.8% working fewer hours when they initially returned to work.

Our patients perceived physical rehabilitation as the most important factor facilitating RTW. This finding is supported Bardgett et al. [11]. Their patients reported improved psychological and physical recovery from postoperative rehabilitation sessions. Those that sought additional rehabilitation reported an improved ability to RTW.

Psychological factors including self-motivation and boredom were important in influencing RTW in our patients. Styron et al. [12] found that patients felt a sense of urgency in returning to work was the most important factor. A similar study by Marcinowski et al. [13] identified ‘keeping faith’ (a combination of determination, trust, and optimism) were important factors in a patient’s rehabilitation although they did not specifically comment on RTW.

Job flexibility enabled some of our patients to RTW. Those who worked remotely, undertook fewer hours or lighter duties had an earlier RTW. These factors allowed these patients to begin working an average of 4.8 weeks after surgery, compared to 6 weeks for the rest of the cohort. Bardgett et al. [14] found that similar employer-facilitated adaptations, phased RTW, work space adaptations and reduced workload facilitated RTW.

The physical effects of surgery (e.g., pain/fatigue) were the main barriers to RTW in our study. All patients in a study by Maillette et al. [15] who did not RTW after TKA reported more pain than those who did RTW.

Work-related factors impeded some of our patients returning to work. Bardgett et al. [14] similarly identified that the absence of a phased RTW, work space adaptations and support was associated with a negative experience of returning to work in arthroplasty patients.

Sankar et al. [3] found that patients who returned to work at 1 month had more pain and more functional/work limitations than those who returned to work later. This implies that they may have returned to work too early and may have benefitted from a graduated RTW.

Medical advice restricting patients to RTW was a common factor. Most patients in our study returned to work approximately 6 weeks postoperatively. This correlates with the first postoperative outpatient review. Most patients stated that the operating surgeon was their primary source of advice regarding returning to work. It is likely that most patients were permitted to resume work at this stage. Bardgett et al. [14] noted that advice from healthcare professionals was considered inconsistent and not tailored to the patient’s needs. Another study highlighted that returning to work was not discussed preoperatively leading to uncertainty amongst patients [11]. Such comments were not noted in our study, probably because the operating surgeon saw all patients at each visit, leading to consistency of advice.

Limitations of this study were the small sample size, which limited the power of the quantitative results. A patient-completed survey was used to collect the qualitative data. This did not allow potentially ambiguous responses to be clarified. There was no control group. Patients were not randomised. The cohort of patients surveyed was limited to individuals with private health insurance, and who were residing in a metropolitan area. There was no subgroup analysis based on prosthesis model or design for THA, TKA or UKA.

The strength of this study was that it provided quantitative data on when patients returned to work, and the nature of work performed after hip and knee arthroplasty. It also provided patient perspectives on what influenced their RTW.

In conclusion, the time of RTW was not significantly influenced by the operation type, or by the physical demands of the job. This study identified that 91% of patients achieve their goal of resuming employment, at a median time of 6 weeks following their joint arthroplasty. The key factors that positively influenced RTW were rehabilitation, desire, necessity and job flexibility. Pain, fatigue, medico-legal restrictions and inability to perform work duties negatively influenced patients returning to work after hip and knee arthroplasty.
